# Bimodal Tactile Tomography with Bayesian Sequential Palpation for Intracavitary Microstructure Profiling and Segmentation

**DOI:** 10.34133/cbsystems.0348

**Published:** 2025-09-02

**Authors:** Wenchao Yue, Chao Xu, Tao Zhang, Jianing Qiu, Wu Yuan, Hongliang Ren

**Affiliations:** ^1^ Department of Electronic Engineering, The Chinese University of Hong Kong, Hong Kong SAR, China.; ^2^ Department of Biomedical Engineering, The Chinese University of Hong Kong, Hong Kong SAR, China.

## Abstract

Robotic palpation for in situ tissue biomechanical evaluation is crucial for disease diagnosis, especially in luminal organs. However, acquiring real-time information about the tissue’s interaction state and physical characteristics remains a substantial challenge. While commercial surgical robotic systems have integrated tactile feedback, the absence of tactile intelligence and autonomous decision-making limits the surgeon’s ability to comprehensively assess tissue mechanics, hindering the efficient detection of abnormalities. Endoscopic optical coherence tomography has emerged as a promising technology for real-time, 3-dimensional visualization of tissue microstructures and subtle lesions in luminal organs. However, it does not address the tactile sensing required for lesion profiling and boundary identification. To bridge this gap, we developed a new robotic bimodal palpation technique that uses a previously proposed optical-coherence-tomography-based tactile sensor, ElastoSight. This technique utilizes circumferential and sliding B-scan modes along with Bayesian optimization for precise lesion center and boundary detection. In tumor phantom models, our technique achieves tumor localization within 30 iterations, with high F_1_ scores over 0.976 and a centroid error below 0.032 mm. Using the sliding B-scan mode on the phantom surface, we achieve accurate segmentation of hard tissue inclusions from the surrounding soft tissue, with a precision rate of 0.983 and an area error below 0.25 mm^2^. These results show that the proposed technique effectively tackles real-time lesion localization and segmentation challenges, demonstrating strong performance in simulations and experiments. Our technique can potentially enhance tissue abnormality detection during robot-assisted minimally invasive surgery, improving the precision and efficiency of procedures like tumor removal.

## Introduction

Robot-assisted minimally invasive surgery (RMIS) combines the advantages provided by surgical robots, such as tremor filtration, reduced muscle fatigue, 3-dimensional (3D) vision, and improved eye–hand coordination [1,2]. Surgeons can teleoperate with medical robots in RMIS to perform complex and delicate surgical procedures. Intraoperative feedback enhances surgical immersion, improves success rates, reduces cognitive fatigue, and offers other notable benefits [3–5].

Most commercial RMIS systems incorporate endoscopic cameras or other visual feedback tools, such as fluorescence imaging, to assist surgeons in controlling end-effector tools [6]. Although the current fifth-generation da Vinci system implements force feedback, it still lacks decision-making algorithms to capture high-dimensional and high-fidelity tactile information. Meanwhile, the limited endoscopic view significantly constrains the operation [7]. Additionally, intraoperative tissue deformation limits the additional information of preoperative imaging technologies like computed tomography and magnetic resonance imaging [8]. These technologies also cannot precisely resolve and locate buried hard inclusions with a diameter of less than 1 mm. In addition to visual feedback, incorporating tactile feedback provides valuable information about the biomechanical properties of tissues [9], which enables the identification of blood vessels and buried hard inclusions, such as tumors and lumps. Various tactile sensors have been proposed to monitor distal force during surgery to reconstruct interaction information between the end effector and tissue, including piezoresistive sensors [10,11], piezoelectric sensors [12–15], capacitive sensors [16], strain gauge sensors [17], and fiber-Bragg-grating-based sensors [18,19]. Although these technologies can detect stiffness gradients in tumors, they operate as a single modality, which may not ensure the high fidelity of the acquired signals. The limited visibility of tumors using single-modal inputs, such as white-light vision, is an unsolved challenge in early tumor detection [20–23]. Recently, there has been a surge of interest in using the optical coherence tomography (OCT) technique as a noninvasive method for evaluating a tissue’s stiffness of early tumor [24,25]. The concept and prototype of deploying intracavitary optical coherence elastography into robotic systems have also been proposed and investigated [26]. While our previous work primarily focused on the noncontact inspection of OCT systems integrated with robotics, it did not address the estimation of tissue stiffness or the autonomous acquisition of additional information within the target tissue [27–29]. Implementing these capabilities would enhance the comprehensive information available for more precise tissue diagnosis.

Tissue stiffness mapping can be achieved through various methods. The most prevalent technique involves discrete probing, where a tactile sensor mounted on a robotic arm systematically samples the tissue surface at defined points on a dense grid [30]. However, this exhaustive palpation process is often inefficient due to the significant time required for thorough exploration. An alternative approach involves framing the localization of tissue abnormalities as a Gaussian process regression (GPR) problem [31]. This method aims to identify regions of maximum stiffness based on real-time stiffness distribution estimates derived from sensor data. Some adaptive probing strategies have been developed to enhance exploration efficiency by prioritizing regions with high stiffness gradients, especially near estimated tumor boundaries [32–34]. In contrast to discrete probing methods, some approaches utilize continuous motion to evaluate tissue properties [35,36]. Continuous cycloidal motion combined with adaptive search algorithms has significantly reduced the overall exploration time [37]. It is worthwhile to note that the segmentation-related work aligns closely with the field of sliding tactile sensing. Some studies utilize Hall effect-based tactile sensors for sliding tactile sensing, which can effectively record the hardness distribution of tissues along the path when sweeping the surface of the target area [32]. By employing GPR, these methods actively select the sweeping path, allowing for the generation of a hardness distribution image after multiple iterations. Additionally, palpation-based imaging systems based on force sensors and GPR can align the target geometry with preoperative models, enabling rapid localization of areas with abnormal hardness through continuous sweep sampling [38]. To leverage the advantages of discrete and continuous sampling methods, a hybrid strategy has been developed utilizing a soft magnetic tactile sensor [39]. This approach begins with discrete adaptive sampling to accurately estimate the lesion’s center, followed by sliding segmentation to profile the tumor’s shape. However, the risk of tissue abrasion during the sliding process poses significant challenges for its clinical application. Addressing this concern is essential for improving the technique’s safety and effectiveness in surgical settings.

**Fig. 1.**
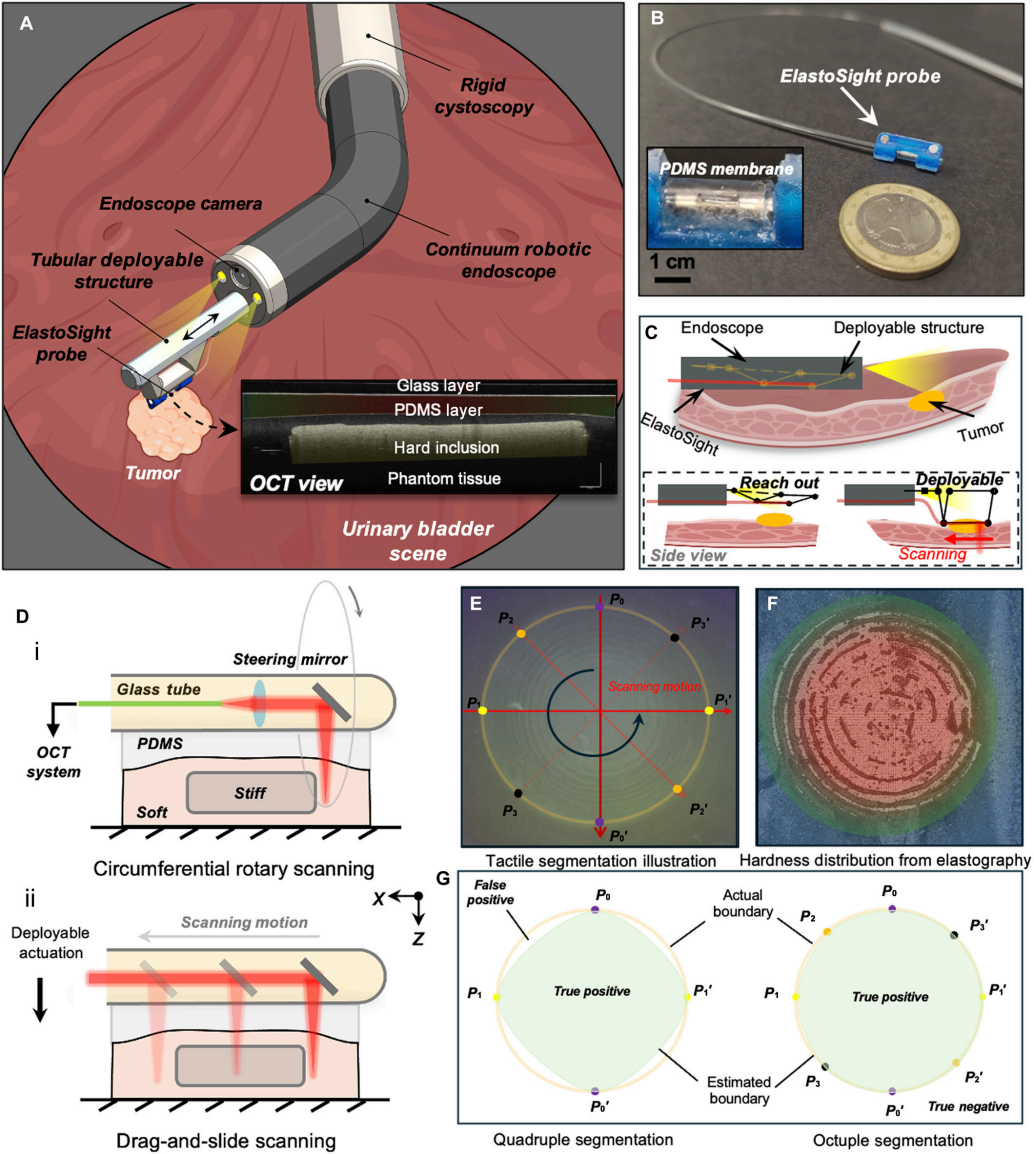
Working principle of bimodal tactile tomography for intracavitary microstructure profiling and segmentation. (A) Illustration of a distally mounted robotic endoscopic module integrated with the deployable ElastoSight probe for proprioceptive shape observation and force sensing. Schematics of the sliding palpation setup for the tactile segmentation of the inclusion phantom. The scale bar in the figure represents 250 μm. (B) To-scale ElastoSight probe with a €1 coin for scale. (C) Deployable demonstration for active palpation application. (D) The working principle of dual-modal distal B-scan imaging measurement: (i) One modality involves circumferential rotary scanning, achieved through motor-driven rotation, primarily utilized for active palpation to estimate the central location of tumor points. (ii) The other modality employs drag-and-slide scanning, realized through motorized dragging motion, primarily aimed at tumor shape segmentation and identification. (E and F) Schematics of active sliding tactile segmentation toward circle tumor phantom from the raw and elastography views. (G) Compared with the actual boundary, the estimated tumor boundary shape using quadruple and octuple segmentation. OCT, optical coherence tomography; PDMS, polydimethylsiloxane.

To address the limitations of the current work, we propose a novel and more efficient hybrid sampling strategy that integrates multiple Gaussian active-guided discrete sampling methods to accurately determine the lesion’s center. This approach capitalizes on the inherent strengths of OCT-based tactile sensors, which facilitate frictionless sliding while ensuring continuous contact with the tissue. By combining these techniques, our strategy aims to enhance the precision of tumor localization and shape estimation, ultimately improving the safety and effectiveness of surgical interventions, demonstrating great potential for robotic intraluminal palpation, as shown in Fig. 1A. The palpation output result depicts a cross-sectional OCT view of the phantom tissue for component identification and boundary point detection. The proposed view can clarify the contact state by revealing parameters such as contact force and relative stiffness. It also allows for identifying different materials, including the components of ElastoSight [40] (the glass and polydimethylsiloxane [PDMS] layers) and the tumor phantom (the hard inclusion layer and phantom tissue). To sum up, this work makes 3 key contributions:

• We introduce a hybrid sampling technique that utilizes OCT-based tactile sensing. This method combines dual-mode distal B-scan imaging measurement, including circumferential and sliding B-scan modes, to actively locate lesion centers and enable programmable boundary segmentation, enhancing the task-specific computational complexity reduction to 6,249-fold, lowering the center error to 0.032 mm, and improving the shape fitting accuracy to 0.983.
• We present a variable-resolution approach within a grid-based discrete search framework tailored for rotation B scanning. This involves comparing 6 different sampling strategies to effectively estimate the centroid of tumor phantoms, offering insights for improved localization with a higher spatial resolution of 10*x* times (*x* = 1, 2, 3…) in grid sample space.
• We propose and thoroughly assess a programmable tactile segmentation strategy using the sliding B mode. This comprehensive evaluation demonstrates the efficacy of our proposed approach and its potential applications in real-time tissue analysis, contributing to advancements in minimally invasive procedures.

## Methods

### Overview of the perception principle

To enable surgeons to obtain more information through the robot in a noncontact or even invisible state, we need to synthesize the use of OCT-based tactile sensors in Fig. 1B to more comprehensively and efficiently obtain the hardness information of the target area using robotic palpation in Movie S1. Fig. 1C illustrates a clearer demonstration of the procedure: the ElastoSight probe is deployed to enable active palpation toward the lesion regions. During this process, the probe is inserted through an endoscope to approach and detect tumors in 2 stages: “reach out” and “deployable”. In the “reach out” stage, the deployable devices attached to ElastoSight extend from the endoscope toward the tumor. In the “deployable” stage, the structure contacts the tumor and performs “scanning” imaging. This method enhances the probe’s accuracy and provides more detailed information about the lesion regions, aiding further diagnosis and treatment. Therefore, we aim to quickly localize the tumors and find their precise outline via autonomous robotic palpation. We divide the tumor detection task into 2 subtasks, as shown in Fig. 1D: (I) to utilize the rotation scanning B mode to find the tumor centroid and (II) to utilize the sliding scanning B mode to find the tumor boundary. The robotic tactile system comprises a continuum endoscope alongside an optical coherence elastography stiffness sensor integrated through the endoscopic channel. During the palpation process, the endoscope exhibits 4 degrees of freedom, which include axial rotation, axial feed motion, and bidirectional bending. Coupled with the sliding palpation setup, this system can effectively perform palpation tasks on most of the inner surfaces of the target sample regions.

To find the tumor’s boundary shape, we can also take advantage of the sliding B mode of OCT so that the sensor can explicitly identify the boundary points when sliding over the tumor phantom. Therefore, a sliding B-mode scan can detect boundary points $P_{1}$ and $P_{1}^{\prime }$, providing the basis for further tumor shape segmentation. Take a circular tumor as an example shown in Fig. 1E and hardness distribution shown in Fig. 1F; when the sensor slides over the circular tumor along 4 directions passing through its centroid, the area of the fitted tumor contour is gradually approaching the real one in Fig. 1G. The following sections provide a detailed description of the sensor sampling strategy. This includes the rotation B scanning mode for measuring relative stiffness and force and the sliding B scanning mode for determining boundary points. Next, methods for active centroid estimation of the tumor phantom based on Gaussian adaptive sampling are discussed. Finally, the programmable sliding strategies for tumor phantom shape segmentation are further introduced and discussed.

### Perception criteria of the OCT-based tactile sensor

**Fig. 2.**
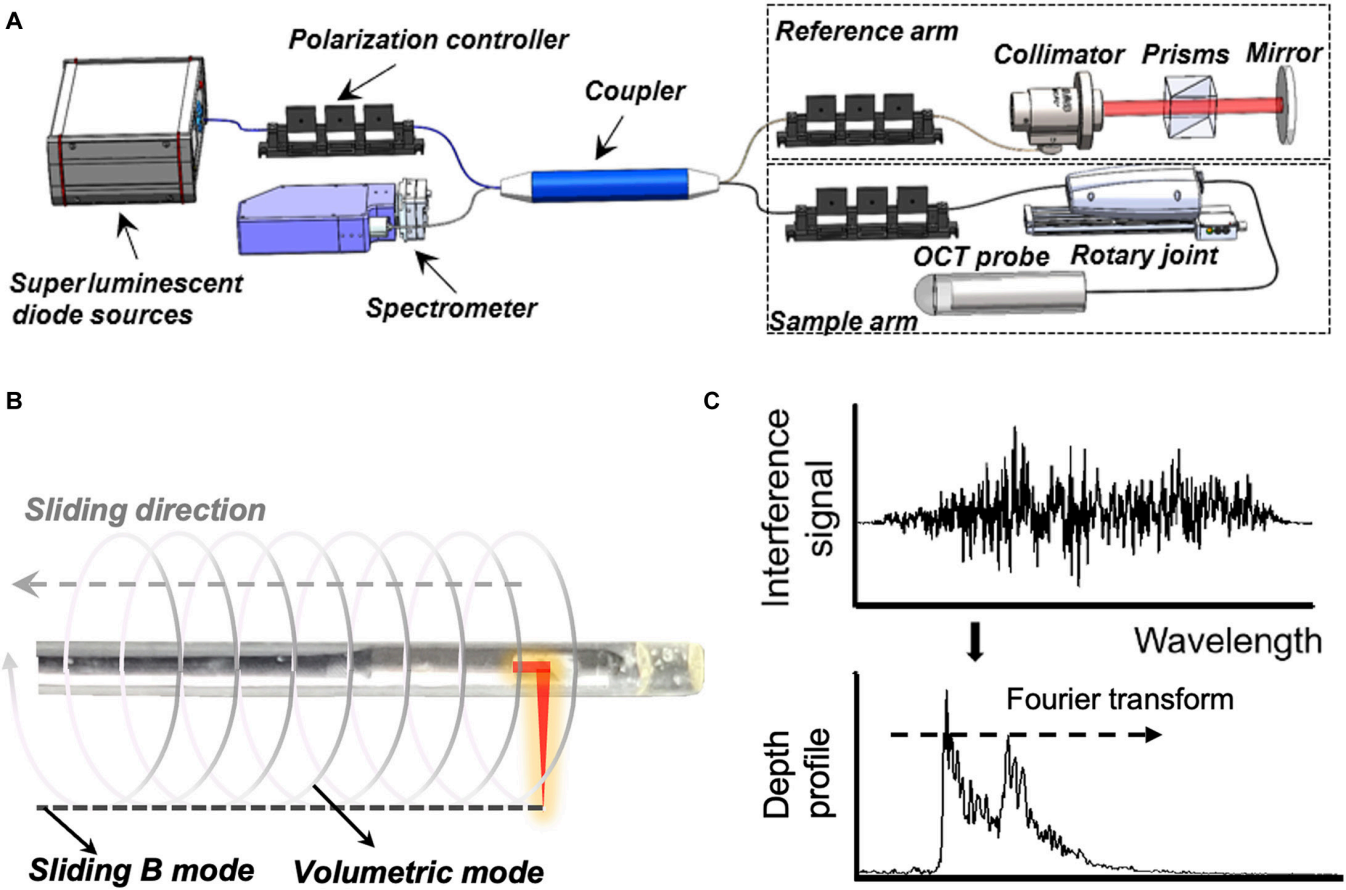
Dual-mode principle of the OCT-based tactile sensor. (A) Schematic and components of the spectral-domain optical coherence tomography (SD-OCT) imaging system. (B) Segmentation principle using the sliding B mode. The sliding B mode and the volumetric mode were compared to prove the data efficiency. (C) The spectral interferograms are acquired with a linear *k*-spectrometer in the SD-OCT system. The depth profile (A line) was reconstructed by taking the Fourier transform of the spectral interferogram.

Sensible input of tactile information is essential for robotic searches within cavities. This critical requirement enables the robotic system to accurately perceive and interpret tactile feedback from its surroundings, facilitating precise navigation and interaction. Accurately determining the actual hardness of tissue presents significant challenges. In our study, we rely on comparative analysis rather than direct measurement. Specifically, we assess the relative thickness of the compressed PDMS film, using previously characterized PDMS samples with known hardness values. By controlling for a consistent contact force during these measurements, we can effectively evaluate the relative hardness changes, allowing the sensor’s adaptation among the different tissue characteristics while acknowledging the limitations inherent in our approach for acquiring ground truth stiffness or actual stiffness. A custom spectral-domain OCT endoscopy system (Fig. 2A) was constructed in this work to image the deformation of the elastic membrane. The configuration was similar to those we developed previously [41]. Specifically, the system used a superluminescent diode light source (MT-850-HP, Superlum Inc.) with a central wavelength of 842 nm and a spectral width of 160 nm. The power of the laser output was 15.6 mW, which was directed to a 50:50 fiber coupler (TW850R5A2, Thorlabs Inc.) via a single-mode fiber (780-HP, Thorlabs) and was split into the sample and reference arms. The reference arm employed a pair of N-SF11 prisms (Edmund Inc.) to compensate for the dispersion imbalance between the reference and sample arms. A custom rotary joint with a linear translation stage (X-LSM150A, Zaber Technologies) performed volumetric imaging in the sample arm. An OCT probe with an outer diameter of 1.8 mm was connected to the rotary joint. The output power of the probe was measured to be 4.2 mW in the study. A spectrometer (Cobra-S 800, Wasatch Photonics Inc.) with a high line-scan rate of up to 250 kHz was used to collect OCT signals. The system provides an axial resolution of 2.7 μm and a scanning depth of 1.05 mm with depth sampling at 0.37 μm per pixel in the membrane (*n* = 1.40). The actual sampling time for a single B scan is influenced by 2 main factors: the rotation speed of the motor and the dragging speed. The linear motor’s dragging speed ranges from 100 to 400 μm/s, while the rotation speed is 40 revolutions per second. The actual sampling time can be estimated based on the sliding distance and the number of sampling points; however, it is also affected by delays associated with the robot’s movement and control. In this paper, we consider only the operational time of the decision and execution phases under ideal conditions.

To better understand the sensor’s performance, we first modeled the contact state during the calibration process. This modeling was essential for experimentally verifying the sensor’s functionality. By clearly representing the contact dynamics, we aimed to identify key factors affecting the sensor’s responsiveness and accuracy. The subsequent experimental validation allowed us to evaluate both the model’s reliability and the sensor’s effectiveness in practical applications. This section focuses on modeling the interaction state between the calibration probe and a soft membrane in terms of contact geometry and mechanics. For simplicity, the probe tip in this study is spherical; however, it is not restricted to this shape and can be varied as needed, and the Hertz contact theory can be used to analyze the measured displacement and estimate contact force [42]. To simplify the problem, the contact area’s significant dimensions must be small compared with (1) the dimensions of the force calibration probe and ElastoSight and (2) the relative radii of surface curvature. The surfaces are assumed to be applied by only a normal pressure, and the calibration probe is an infinitely rigid body compared to the soft PDMS membrane of ElastoSight’s surface. The OCT scanning mode used by our proposed ElastoSight is the rotation B-scan mode, i.e., a 2-dimensional cross-section image formed by circumferentially sweeping the surroundings.

The contact force between the object and probe requires proper estimation for continuous mechanical analysis. With the radii $r_{1}$ and $r_{2}$ of the 2 objects, the contact force $F_{N}$ follows the relation with deflection d:

$$F_{N}=Md^{\frac{3}{2}}$$ (1)

where *d* refers to the local relative deflection between the calibration probe and the ElastoSight probe. M denotes the universal parameter, which synthesizes the description of the material property and radius value as

$$M=\frac{4}{3\left(h_{1}+h_{2}+h_{3}\right)}\underset{R_{e}^{\frac{1}{2}}}{\underbrace{{\left[\frac{r_{1}r_{2}}{r_{1}+r_{2}}\right]}^{\frac{1}{2}}}}$$ (2)

where the material parameters $h_{i}$ follow the definition

$$h_{i}=\frac{1-\nu_{i}^{2}}{E_{i}},\;i=1,2,3$$ (3)

where $\nu_{i}$ and $E_{i}$ are respectively the Poisson’s ratios and Young’s modulus associated with the calibration probe, PDMS membrane, and Pebax tube and $R_{e}$ refers to the equivalent radius. It should be noted that the calibration probe is made from the photopolymer with a Young’s modulus of 10^9^ Pa. The Pebax tube has a Young’s modulus in the range of 10^10^ Pa, which is much larger than that of the PDMS membrane in the range of 10^6^ Pa. This indicates that the $h_{1}$ term of the calibration probe and the $h_{3}$ of the Pebax tube have less significant influence, so the $h_{2}$ term of the PDMS film is the dominant term toward the contact force estimation.

### Active search for the tumor centroid based on Gaussian adaptive sampling

To implement an active search strategy, we discretize the target space into a grid and further subdivide the regions to enhance the resolution of the search results, thereby improving lesion localization accuracy. The assumption is that the robot’s workspace is sufficiently designed to reach the specified target area S. In the following formula, $\left\{\;G_{\mathrm{ij}}\;\right\}$ represents the grid point set $\left(x_{i},y_{i}\right)$ covering S, which follows the definition as below:

$$\begin{array}{c} \left\{\;G_{\mathrm{ij}}\;\right\}=\left\{\left(x_{i},y_{j}\right)|x_{i}=x_{0}+i\cdot \Delta x,y_{i}=y_{0}+j\cdot \Delta y\right\} \end{array}$$ (4)

where *i* and *j* are the indices of the rows and columns, respectively. $x_{0}$ and $y_{0}$ are the starting *x* coordinate and *y* coordinate of the grid. Δx and Δy are the interval values between grid points in the *x* direction and *y* direction:

$$\Delta x=\frac{x_{\max}-x_{\min}}{N}$$ (5)

$$\Delta y=\frac{y_{\max}-y_{\min}}{N}$$ (6)

where *N* represents the resolution of the subdivision grid. $x_{\max}$ and $x_{\min}$ are the upper and lower boundaries of the *x* coordinate within the grid area, respectively, and $y_{\max}$ and $y_{\min}$ are the upper and lower boundaries of the *y* coordinate within the grid area, respectively. This diagram depicts the fine and coarse grids and the palpation procedure within the sample area. It showcases the discretization and variable-resolution resampling, presenting coarse grid points (5 × 5) and fine grid points (50 × 50), emphasizing a spatial resolution enhancement of up to 10× for detailed analysis.

The stiffness map estimate $S\left(x\right)$ of the discrete grid space $\left\{\;G_{\mathrm{ij}}\;\right\}$ is modeled as a Gaussian process (GP), initialized with measurements collected from randomly selected locations. The GP is characterized by a mean function $\mu \left(x\right)$ and a covariance function $k\left(x,\;x^{\prime }\right)$, where $x,x^{\prime }\in R^{n}$. Using the current estimate $f\left(x\right)$ and its associated uncertainty, new measurement locations $x_{t+1}$ are iteratively selected based on a defined sampling criterion (e.g., expected improvement) to refine the estimate. These measurements are incorporated into the dataset $D_{t}={\left\{\left(x_{i},f\left(x_{i}\right)\right)\right\}}_{i=1}^{t}$, where $f\left(x_{i}\right)$ denotes the observed force measurements, allowing for retraining of the GP. In the context of tumor detection, we model the stiffness distribution of the tissue as a smooth but unknown function $f:R^{n}\to R$. Observations are available only at specific sampled locations through time-intensive palpation. The GP framework enhances exploration efficiency, facilitating rapid localization of the tumor, thereby transforming the optimization problem into identifying the region of maximum stiffness:

$$x_{\text{tumor}}=\text{arg}\underset{x}{\text{max}}S\left(x\right)$$ (7)

Let the set of observations be defined as $D_{t}={\left\{x_{i},f\left(x_{i}\right)\right\}}_{i=1}^{t}$, where $x_{i}$ are the sampling points on the tissue surface and $f\left(x_{i}\right)$ are the corresponding force measurements obtained from the OCT-based tactile sensor. Given a new input $x_{t+1}$ suggested by the acquisition function, the posterior distribution of the target function value $f\left(x_{t+1}\right)$ can be computed using

$$f\left(x_{t+1}\right)∼N\left(\mu_{t+1},\;\sigma_{t+1}^{2}\right)$$ (8)

where $\mu_{t+1}$ and $\sigma_{t+1}^{2}$ are derived from the GP prior and the observed data, given by

$$\mu_{t+1}=k\left(x_{t+1},\;X\right){\left(K+\sigma^{2}I\right)}^{-1}f\left(X\right)$$ (9)

$$\sigma_{t+1}^{2}=k\left(x_{t+1},\;x_{t+1}\right)-k\left(x_{t+1},X\right){\left(K+\sigma^{2}I\right)}^{-1}k\left(X,\;x_{t+1}\right)$$ (10)

where $X={\left[x_{1},\ldots ,\;x_{t}\right]}^{T}$ is the matrix of observed locations, K is the covariance matrix of the observed outputs, and $\sigma^{2}$ represents the noise variance. The covariance function 𝑘 determines the correlation between input locations $x_{i}$. We use the squared exponential kernel for the experiments in this paper because it produces smooth output:

$$k\left(x_{i},\;x_{j}\right)=\sigma^{2}\text{exp}\left(\frac{-{\left\|x_{i}-x_{j}\right\|}^{2}}{2l^{2}}\right)$$ (11)

where $\sigma^{2}$ is the variance of the kernel and l is the hyperparameter that controls the width of the kernel. We have established the objective function for the stiffness distribution using a GP prior, aiming to optimize this function to identify regions of increased stiffness in tissue. The key challenge is determining the optimal sampling locations within the continuous search space to locate the global maximum indicative of a tumor effectively. In this study, the search space corresponds to the organ’s surface, where each point is labeled as either normal tissue or abnormal tissue. We investigate various active learning algorithms, including expected variance reduction (EVR) [44], expected improvement (EI) [45], active level sets estimation (LSE) [46], upper confidence bound (UCB) [47], implicit level set upper confidence bound (ILS-UCB) [32], and rescaling acquisition strategy with energy constraints (RASEC) [34]. These methods are compared against a Bayesian optimization algorithm to balance global search and local optimization, enhancing exploration and exploitation.

### Tumor shape segmentation based on the sliding B mode

Although the proposed algorithm can effectively localize the tumor’s centroid, it may still lack precision in segmenting it from the surrounding soft tissue. To address this, we introduce a novel method for tumor segmentation that detects the boundary points of the tumor while sliding the OCT-based tactile sensor across its surface. To determine the number of A lines present in each B scan, we begin by considering key parameters that influence the data acquisition process. The A-line rate is specified as R=250kHz, which translates to 250,000 A lines per second. Additionally, the rotational speed of the catheter is F=40 frames per second (equivalently, 40 rotations per second).

The number of A lines acquired during one complete catheter rotation directly corresponds to the number of A lines in each B scan. This relationship can be expressed mathematically as

$$N_{A\;\text{lines}\;\mathrm{per}\;B\;\text{scan}}=\frac{R}{F}=\frac{250,000\;A\;\text{lines}/s}{40\;\text{frames}/s}=6,250$$ (12)

Thus, in conventional endoscopic OCT, each B scan consists of 6,250 A lines, as determined by the above formula. In contrast, our proposed sliding B-mode method significantly simplifies this process. To achieve this, we integrate the sensor with a sliding B-scan mode, enabling it to capture variations in optical signals $I\left(\lambda \right)$ as it moves over tumor boundaries. Instead of acquiring multiple A lines, this approach requires only a single A line to gather the necessary data for analysis, as shown in Fig. 2B. This modification not only reduces the data burden associated with the imaging process but also enhances the overall efficiency, thereby streamlining the data acquisition in endoscopic OCT applications. When the sensor traverses the first tumor (located on the left side of the tissue) along 4 directions passing through its centroid C, the measurements of the optical signals exhibit significant changes near the tumor boundaries based on the principle from our previous work [20] shown in Fig. 2C. For instance, when the sensor slides across the tumor from $P_{1}$ to $P_{1}^{\prime }$ along the y axis (sliding 1), we observe a substantial increase in the optical signal $I\left(\lambda \right)$ at $P_{1}$ followed by a marked decrease at $P_{1}^{\prime }$. This behavior occurs because the stiffness $k_{t}$ of the tumor (including points $P_{1}$ and $P_{1}^{\prime }$) exceeds that of the surrounding soft tissue $k_{s}$, resulting in greater deformation Δz of the sensor along the z axis. In contrast to the monotonous changes observed in the optical signal at each boundary point, the signal exhibits 2 distinct changes at $P_{1}$ and $P_{1}^{\prime }$ due to lateral sensor deformation. Specifically, when passing $P_{1}$, the sensor deforms on one side, while it deforms on the opposite side when passing $P_{1}^{\prime }$. This results in opposing deformation directions on either side of the sensor, leading to spike-like signals with opposite polarities: a negative pulse $P_{-}\left(P_{1}\right)$ at $P_{1}$ and a positive pulse $P_{+}\left(P_{1}^{\prime }\right)$ at $P_{1}^{\prime }$. To clarify further, when the pulse interacts with a hard block, it experiences a leftward shift, which we categorize as a negative pulse $P_{-}\left(P_{1}\right)$. This phenomenon indicates that the peak of the pulse is displaced toward the left side of the baseline. In contrast, the positive pulse $P_{+}\left(P_{1}^{\prime }\right)$ is characterized by a rightward bias, where the peak shifts to the right. Consequently, the 2 boundary points along the sliding trajectory correspond to the 2 pulse peaks $\left\{P_{-}\left(P_{1}\right),\;P_{+}\left(P_{1}^{\prime }\right)\right\}$ in the optical signal curve, which can be readily extracted for tumor segmentation. However, the starting point and the length of the sliding trajectories are manually predetermined, which is impractical for real applications where the shape and size of the tumor are unknown. A more effective approach involves sliding the OCT-based tactile sensor from the centroid C of the tumor outward to detect only one boundary point P at a time rather than 2. This method is feasible since we can identify the tumor centroid C beforehand and halt the sliding process upon detecting a spike in the optical signal measurements $I\left(\lambda \right)$.

By initiating the sensor’s motion from C in various directions defined by angles $\theta_{i}$, we can record a series of tumor boundary points $\left\{P_{i}\right\}$ corresponding to the detected spikes. As we slide the sensor outward in a direction θ, we monitor the changes in $I\left(\lambda \right)$ until a significant increase ΔI is observed, indicating a boundary point $P_{i}$. This allows us to effectively gather a comprehensive set of boundary points, which can then be interpolated to outline the tumor’s shape. Thus, the algorithm not only improves the practicality of the segmentation process but also enhances the accuracy of tumor boundary detection by leveraging the optical signals captured during the sliding procedure while also addressing potential tissue abrasion caused by friction in previous studies and mitigating segmentation errors due to hysteresis in electromagnetic signals.

## Results

### Sensing performance experiment

**Fig. 3.**
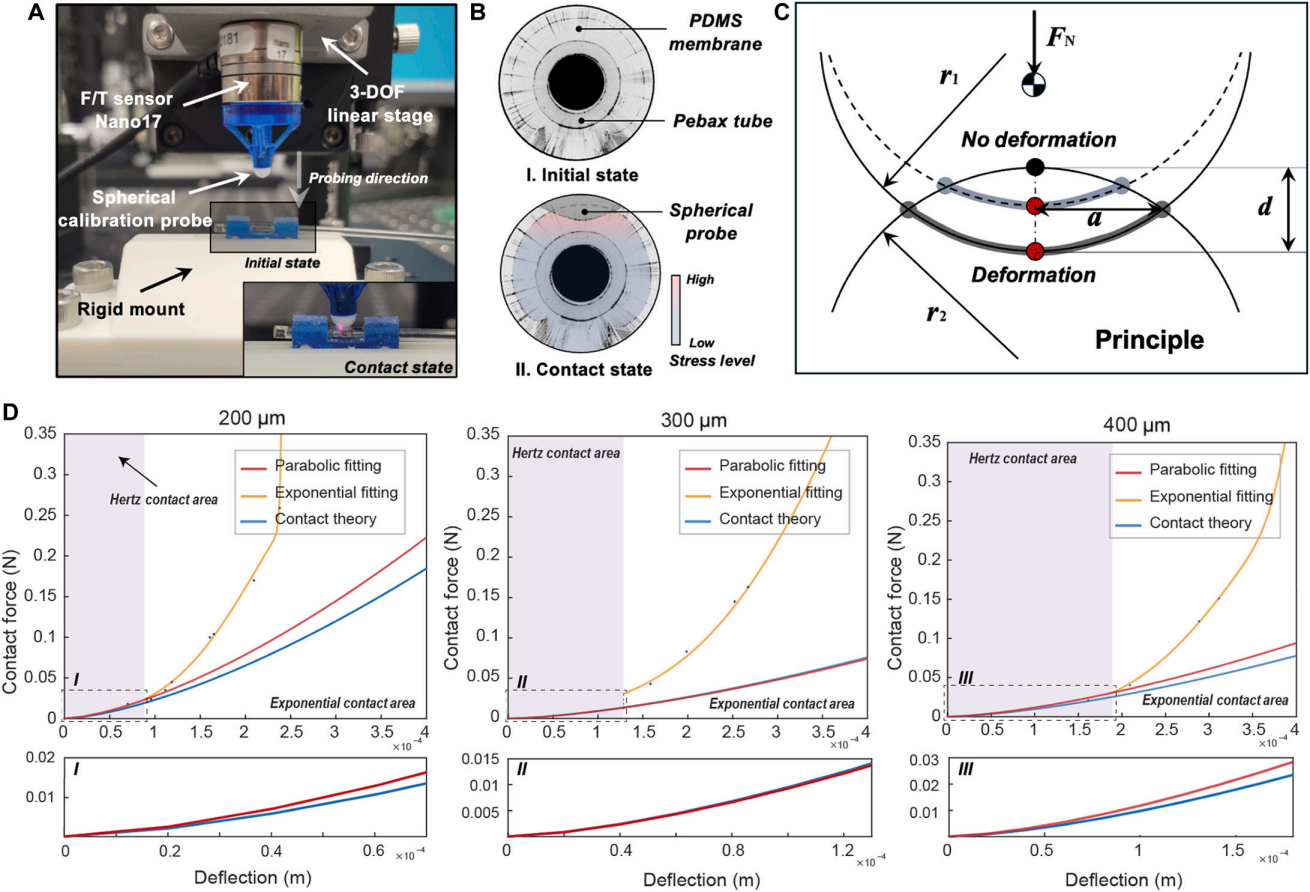
Experimental results of shape estimation and force calibration for the ElastoSight sensor. (A) Calibration experiment setup. (B) Contact state and deformation observation of the ElastoSight probe. (C) Schematic of the rigid calibration probe in contact with the elastic surface of the ElastoSight probe. (D) Normal deflection distribution of PDMS membranes (200-, 300-, and 400-μm thicknesses) under normal loading (0 to 0.3 N). F/T, force/torque; DOF, degree of freedom.

The link between the actual deformation of the PDMS film and the contact force can be affected by the force signal noise, light intensity, refractive index, and OCT imaging parameters. Therefore, we can calibrate this relationship between contact force and the film deflection against ground truth. In the calibration process, an ATI Nano17 sensor (ATI Industrial Automation, Apex, NC, USA) with a customized 3-degree-of-freedom linear stage is utilized to measure the contact forces, and the spectral-domain OCT probe observes the contact deflection in Fig. 3A. The state of the PDMS film is observed to determine the deflection distribution across the contact area shown in Fig. 3B and C. In contrast, the measured contact force and deflection values are recorded to establish the ground truth for mapping the relationship.

During the experiment, we ensured that the calibration contact with no tangential slip was within the scope of the B-scan imaging range, ensuring that the normal loading could be well observed. Given the assumptions underlying Hertz’s contact theory, the contact was maintained as close to frictionless as possible. Calibration results between the normal contact force and the indentation deflection obtained for PDMS membranes are shown in Fig. 3D. Based on the observation, when the deformation is small, the trend of the experimental calibration data is more consistent with the Hertz theoretical contact model. However, as the deformations become significant, the experimental data show exponential growth so that the calibration curve intervals can be divided into the Hertz contact region and the exponential contact region. The calibration curve is divided into 2 areas: one is the Hertz contact area, which follows the parabolic shape, shown in the enlarged views I, II, and III; the other is the exponential contact area, which follows the exponential shape. We have made further optimizations by removing the references to the 200- and 300-μm-thick films, retaining only the 400-μm film in this work. As such, the calibrated fitting equations for contact force and PDMS film deformation in ElastoSight of a 400-μm film are denoted in Eq. (13):

$$\begin{array}{c} {\left[F_{N}\right]}_{400\;\mathrm{\mu m}}=\\ \left\{\begin{array}{cc} 11,751.422d^{\frac{3}{2}}, & 0<d<1.85\times 10^{-4}\\ 0.3622\text{exp}\left(-{\left(\frac{x-4.361\times 10^{-4}}{4.186\times 10^{-5}}\right)}^{2}\right) & \\ +0.3113\text{exp}\left(-{\left(\frac{x-4.651\times 10^{-4}}{1.814\times 10^{-4}}\right)}^{2}\right), & d>1.85\times 10^{-4} \end{array}\right. \end{array}$$ (13)

By dividing the calibration curve into these 2 distinct areas, the different behaviors of the contact force can be accurately captured, providing insights into the transition from Hertz contact to exponential contact as the deflection increases. Based on the results in Fig. 3D, increasing the film thickness directly expands the Hertz contact area, leading to improved measurement stability. However, the wider exponential contact region increases the risk of damage to the OCT probe and introduces additional perturbations to experimental measurements.

**Fig. 4.**
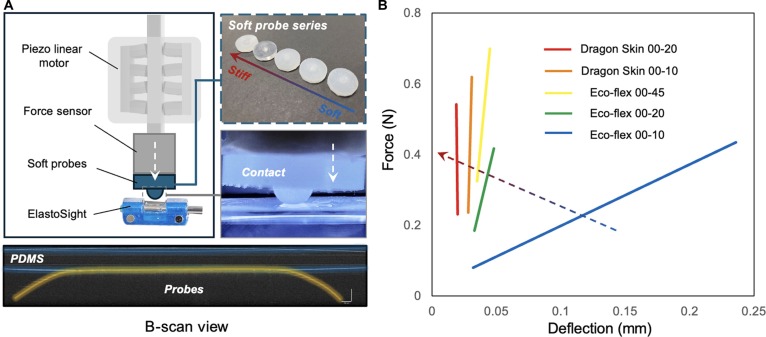
Verification experiment setup and results. (A) The verification experiment setup for our proposed ElastoSight. The scale bar in the figure represents 250 μm. (B) Verification curves of contact force versus sensor deflection using soft silicone probes with 5 different stiffness levels.

To further validate the sensor’s ability to differentiate between hard and soft tissues and provide a solid foundation for future active search, we constructed a verification experiment setup to test soft probes with different stiffness levels, as shown in Fig. 4A. It includes a detailed diagram labeling key components such as the piezo linear motor, ATI force sensor, soft probes, and ElastoSight. Additionally, there are 2 photographs: one illustrating a series of soft probes ranging from stiff to soft and another showing a soft probe in contact with a surface. At the bottom, a B-scan view image displays the probes in contact with a PDMS surface. Fig. 4B shows results from soft probes with different stiffnesses. It presents force (N) against deflection (mm) for various soft probes, i.e., how each material’s force varies with deflection, highlighting their stiffness differences. Five distinct lines in Fig. 4B represent different materials: Dragon Skin 00-20 (red), Dragon Skin 00-10 (orange), Eco-flex 00-45 (yellow), Eco-flex 00-20 (green), and Eco-flex 00-10 (blue). The quantitative verification of force–deflection curves in soft probes offers insights into the mechanical properties of various materials, and this visualization enhances our understanding of how material stiffness affects performance.

### Fruit palpation experiment

**Fig. 5.**
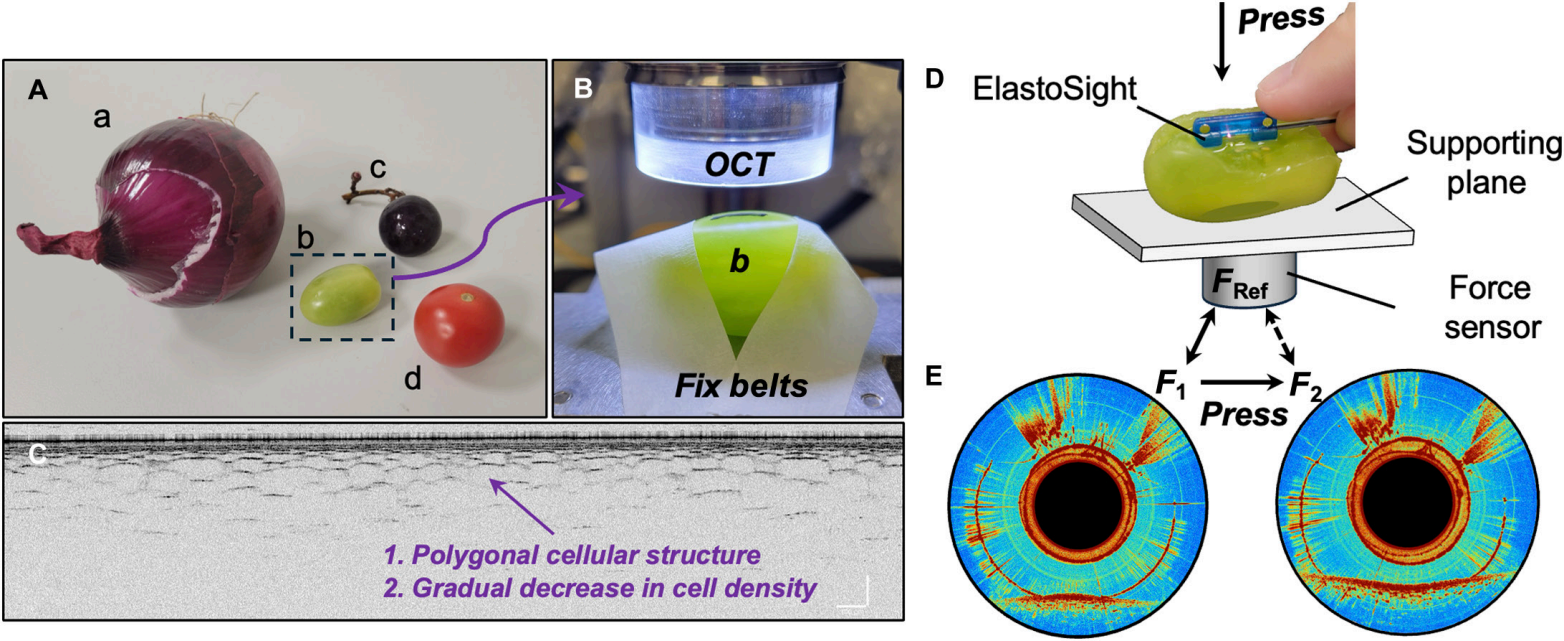
Palpation experiments on fruit phantoms. (A) Four types of test fruits are involved: (a) onion, (b) green cherry tomato, (c) grape, and (d) red cherry tomato. (B) The ground truth view of the OCT observation setup, as exemplified by observation of the (b) green cherry tomato, after the fixed fruit sample, and with a desktop OCT observation of the fruit at the marked position. (C) Cellular geometry. (D) Manual palpation using ElastoSight. (E) Observation view from ElastoSight. The scale bar in the figure represents 250 μm.

In addition to previous experiments validating force and shape using the OCT tactile sensor, we conducted palpation experiments with various fruits to assess the multimodal sensing capabilities of the OCT-based sensor. This section evaluated the sensor’s ability to recognize subcutaneous anatomy within tissues explicitly and to perceive applied contact force concurrently. By utilizing fruits with different textures and densities, we sought to create a diverse testing environment to enhance our understanding of the sensor’s performance in real-world applications. The test objects are listed in Fig. 5A, including a red onion and a green cherry tomato, a black grape, and a red cherry tomato. To accurately represent the subcutaneous skin structure, we used a desktop OCT device (from Thorlabs) to B-scan a fixed fruit sample, taking the example of green cherry tomato in Fig. 5B. The green tomato is secured in a fixture and prepared for an OCT test. Figure 5C shows the cellular structure of the test fruit, highlighting polygonal cell structures and the phenomenon of decreasing cell density. It can allow us to gather precise structural data for comparison when operating the manual palpation. By choosing a fruit phantom with tissue characteristics similar to an anatomical structure, we aimed to establish relevance to evaluate the sensor’s multimodal performance further effectively. Fig. 5D illustrates manual palpation experiments conducted with ElastoSight on a green grape placed on a support plane connected to an ATI force sensor. Figure 5E shows the observation view from ElastoSight, allowing us to compare the estimated forces derived from its observations with the measurements obtained from the ATI force sensors under different applied forces, such as *F*_1_ and *F*_2_. This comparison provides valuable insights into the accuracy and effectiveness of ElastoSight in force estimation during palpation tasks.

**Fig. 6.**
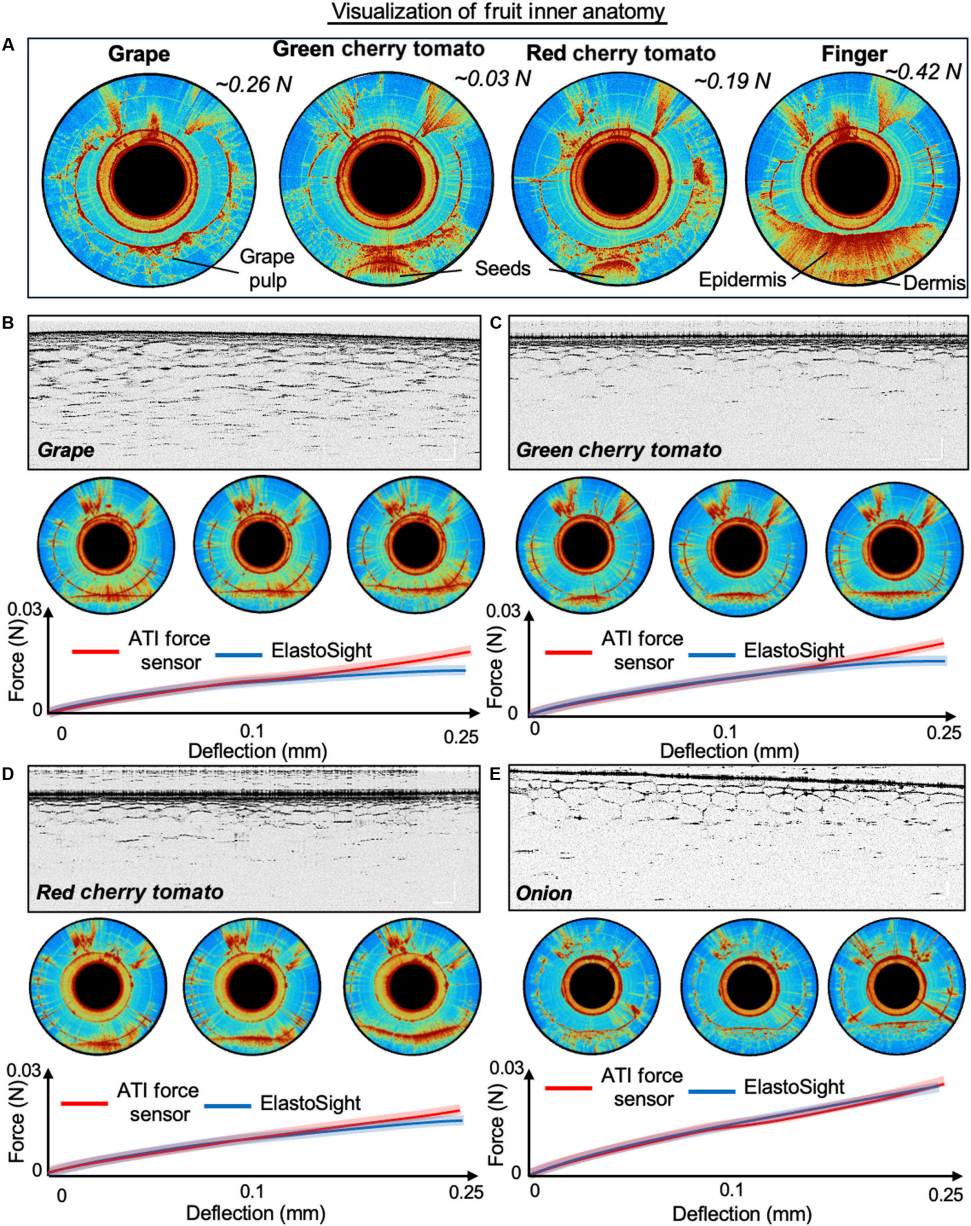
Visualization and quantity results of palpation experiments on fruit phantoms. (A) Visualization results of simultaneous estimation of contact force and internal structure of grapes, tomatoes, and fingers. (B), (C), (D), and (E) illustrate the OCT images as the ground truth, ElastoSight view, and force–deflection curve with a 5% error bar from ATI force sensors and ElastoSight of various fruits. The scale bar in the figure represents 250 μm.

Figure 6A showcases the anatomical structures of various fruits and a human finger with the corresponding force estimation: it presents OCT-based tactile information from the ElastoSight probe obtained under varying applied forces, each annotated with specific force values and details regarding the fruit inside anatomy components in Movie S2. For instance, an applied force of 0.26 N on the grape can be estimated, and the anatomical structure of the grape pulp can also be observed, illustrating the internal characteristics of the fruit. Similarly, the applied forces of 0.03 and 0.19 N on the green and red cherry tomatoes reveal the presence of their seeds, highlighting the internal structure. Under a force of 0.19 N, the red cherry tomato allows for observing the epidermis, indicating its outer layer. Finally, the applied force of 0.42 N on the finger reveals the epidermis and dermis, showcasing the underlying skin’s hierarchical layer. The grayscale images in Fig. 6 illustrate the internal structures labeled grape, green cherry tomato, red cherry tomato, and onion. Each grayscale image is accompanied by force–deflection graphs comparing the readings from 2 sensors: the ATI force sensor (red line) and ElastoSight (blue line). The graphs show force readings with 5% error under different deflections, with the *x* axis representing displacement (mm) ranging from 0 to 0.25 mm and the *y* axis representing force (N) ranging from 0 to 0.03 N. These results show that the OCT-based tactile sensor can detect force and subcutaneous morphology simultaneously, providing quantitative data to evaluate the relative stiffness of tissues in contact. This supports the subsequent implementation of tactile-based active search techniques.

### Centroid estimation results

**Fig. 7.**
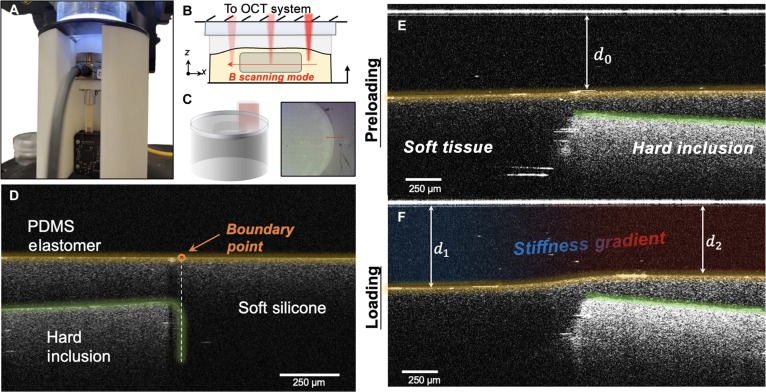
Tumor boundary detection using sliding B mode. (A) Experimental setup for the ideal sliding B-scan mode. (B) Principal diagram of sliding scanning to recognize hard block boundaries. (C) Spatial representation and original camera view during boundary scanning. (D) Observations from the sliding B scan in the unloaded state show a hard inclusion in the phantom. (E) and (F) show that before and after loading, the deformation of the PDMS film assembled by ElastoSight can be observed to estimate the hardness distribution and determine the hard inclusions’ boundary and distribution location.

To effectively demonstrate the utilization of signals from 2 OCT modalities for efficient tactile searching, we established a desktop OCT platform, as illustrated in Fig. 7A, which allows us to conduct the searching task under optimal experimental conditions, providing a robust framework for our investigations. The contact mode employed in our experiment setup closely follows ElastoSight’s perception principle when interacting with the tissue phantom. Specifically, the light beam sequentially traverses a glass layer, followed by a PDMS film layer and, finally, the phantom layer, as illustrated in Fig. 7D. We preloaded the phantom using a piezoelectric linear motor to ensure stable contact during the experiments. This allows us to achieve a consistent hardness distribution, which is crucial for the validity of our contact force calibration and validation experiments. The A scan from the desktop OCT device is analogous to ElastoSight’s rotational B scan, providing deformation information necessary for identifying tumor regions through single-point contact. In contrast, the B scan from the desktop OCT device resembles ElastoSight’s sliding B scan, as shown in Fig.7C, which helps reveal the stiffness distribution and the tumor’s boundaries, allowing for detailed analysis. In Fig. 7E and F, in the unloaded state, the initial thickness of the PDMS film is denoted as *d*_0_, and the thickness distribution remains uniform. Upon loading, when ElastoSight makes contact with the phantom tissues, the region within the hard inclusion becomes noticeably compressed with a thickness of *d*_2_. In contrast, the softer tissue areas exhibit significantly less compression in the *d*_1_ thickness. This differential compression allows for clear observation of the soft tissue’s response, and the location and shape of the hard inclusion within the tissue phantom are distinctly visible. These observations provide an effective and robust validation platform for assessing our algorithm’s ability to locate the tumor center and segment the tumor shape accurately. We further conducted a comprehensive comparison of multiple algorithms on the tumor phantom to validate the effectiveness of various sampling algorithms and enhance the accuracy of estimating the geometric center of the samples, which is crucial for subsequent sliding segmentation.

To validate our method further, we constructed a tissue phantom using silicone and hard inclusions that were 3D printed with PLA, both in a planar geometry with a constant inclusion depth. Additionally, the stiffness map of the tissue phantom is normalized by subtracting the mean and dividing by the maximal peak-to-peak variation. For clarity, the phantom’s hard inclusion is made of 3D-printed PLA with a hardness of approximately 5 GPa, while the soft part consists of Ecoflex 00-10 with a hardness of around 7 kPa. The dimensions of PLA hard inclusion are as follows: 12.57 mm^2^ for the circular sample, 7.79 mm^2^ for the rectangular sample, and 7.88 mm^2^ for the horseshoe-shaped sample, all with a thickness of 0.5 mm. The specified areas of the samples were designed to facilitate desktop OCT observation within its field of view. This consideration ensures that the imaging quality is optimized for accurate analysis. This involved analyzing performance metrics, particularly the F_1_ scores and center estimation results.

**Fig. 8.**
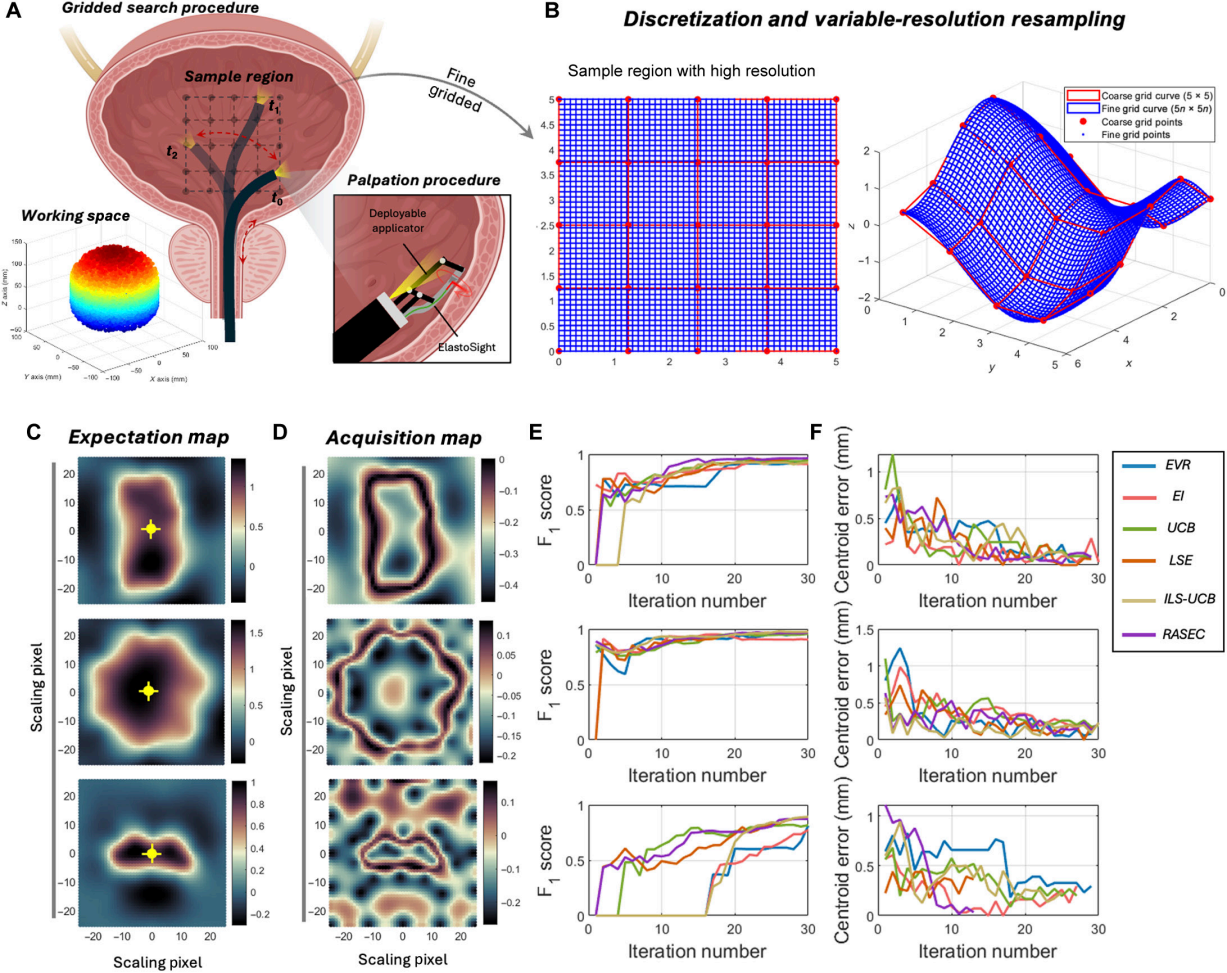
Active estimation of lesion centers using axial rotation B mode. (A) Illustration of the gridded search within the working space and palpation procedure using ElastoSight in a sample region. (B) Discretization and equal interval resampling of the sample region, showing both coarse and fine grid points and curves, aimed at identifying anomalous region centers. (C) Expectation maps for 3 different scaling pixels highlight centroids $C_{1}$, $C_{2}$, and $C_{3}$. (D) Acquisition maps corresponding to the expectation maps. (E) F_1_ score as a function of iteration number for different methods of expected variance reduction (EVR), expected improvement (EI), upper confidence bound (UCB), active level sets estimation (LSE), implicit level set upper confidence bound (ILS-UCB), and rescaling acquisition strategy with energy constraints (RASEC). (F) Centroid error (mm) as a function of iteration number for the same methods.

Fig. 8A depicts the gridded search and palpation procedures within a sample region, demonstrating the use of a deployable applicator with a cable-driven manipulator for transluminal endoscopic surgery and precise localization [43]. The stiffness distribution $K\left(x\right)$ can be used to estimate and locate the tumor’s centroid. To be more specific, Fig. 8B illustrates the discretization process and equal interval resampling of the sample region, presenting both coarse and fine grid points and curves to emphasize the transition from low- to high-resolution sampling, which is crucial for pinpointing the centers of anomalous regions. To relax the need to acquire actual stiffness based on tactile probing, we simplify the tactile search task into a binary classification procedure, and we define the classification threshold using the deflection value based on maximum and minimum deflection (zero in our case) measurements, representing most likely tumor boundaries. In more detail, the criterion for binary hardness classification is based on the observed deflection values of the OCT-based tactile sensor. With the assurance that the contact force remains constant during each interaction, we classify the PDMS film in contact with the tissue by monitoring the changes in its relative thickness. Specifically, we focus on areas where the film undergoes significant compression. This calibration process ensures that our measurements reflect the actual behavior of the PDMS film when in contact with biological tissue, thereby enhancing the reliability of our assessments. The steerable bending length of the endoscope is 95 mm, and the maximum bending angle is 210 deg. The workspace of the endoscope is a cylinder with a diameter of 68.8 mm, shown in Fig. 8A. Here, we outline the classification guidelines: If the acquisition point x is within the hard inclusion region and the tactile sensor’s deflection values exceed the threshold $d_{t}$, the corresponding hardness value y is classified as 1. If the acquisition point x is outside the hard inclusion region and the deflection values are below the threshold $d_{t}$, y is classified as 0. To set the threshold cutoff value at y=0.5, the calibration experiment of the hardness distribution in the target area should be carried out. To achieve accurate classification, we establish a calibrated threshold $d_{t}=150\;\mathrm{\mu m}$ for the thickness of the film in these compressed regions.

Figure 8C shows expected maps at 3 different zoom levels, marked with center points $C_{1}$, $C_{2}$, and $C_{3}$, illustrating the anticipated distribution of the sample characteristics. These maps are followed by their corresponding acquisition maps, which display the actual data collected during the experiment. The final sections of the figure present quantitative analysis results: the F_1_ scores of different methods, such as EVR, EI, UCB, LSE, ILS-UCB, and RASEC, are plotted as a function of the number of iterations, offering insights into the performance and accuracy of each method. Additionally, the center errors in millimeters of the different methods are shown as a function of the number of iterations, indicating the precision of the center point detection in the sample area. Figure 8C and D and Movie S3 visualize the expectation maps and acquisition maps for 3 different scaling pixels, with centroids $C_{1}$, $C_{2}$, and $C_{3}$ marked on the expectation maps. These centroids serve as focal points for analyzing the distribution and intensity of the sampled data, facilitating accurate determination of the centers of anomalous regions.

Figure 8E and F display the performance metrics of various methods used for region identification. Fig. 8E shows the F_1_ score as a function of iteration numbers for 3 types of phantom shapes using different methods, including EVR, EI, UCB, LSE, ILS-UCB, and RASEC. By systematically evaluating these metrics, we aimed to identify the most reliable algorithm for precise geometric center determination, strengthening our analytical framework for handling sample complexities. Figure 8 provides a comprehensive overview of a method used to identify regions of interest within a sample region using ElastoSight, focusing on determining the centers of anomalous regions. As the number of iterations increases, the F_1_ scores for all methods increase and converge to 0.9 at around 10 iterations. The true iteration number here refers to completing a priori sampling until the first valid sampling point of the lesion area is obtained. Figure 8F presents the centroid error (mm) as a function of iteration number during the active search procedure. Based on the average of the 3 centroid active search results, we performed a comprehensive quantitative analysis comparing the performance of different methods: EVR, EI, UCB, LSE, ILS-UCB, and RASEC. Table 1 presents key performance metrics such as F_1_ score and centroid error with the corresponding number of iterations for each acquisition method. Regarding the F_1_ scores, EVR leads with a score of 0.89, achieving the best overall performance, followed by EI and UCB with scores of 0.82 and 0.80, respectively. LSE, ILS-UCB, and RASEC trail behind, with scores below 0.70. In centroid error analysis, EVR achieves the lowest centroid error at 0.20 mm, demonstrating superior precision in detecting the exact location of lesions. EI and UCB have slightly higher errors at 0.35 and 0.38 mm, respectively. The remaining methods exhibit errors above 0.55 mm, indicating less accurate localization.

**Table 1.** Average results on active search for the centroid estimation of phantom models with different shapes in 30 sampling steps. *N* denotes the sampling step, which is highly correlated with the time cost in the actual sampling process. Bold values indicate the best performance (highest F1 score or lowest centroid error) for the corresponding phantom shape group and sample patch.

Phantom	Method	F_1_ score						Centroid error/mm					
		*N* = 5	*N* = 10	*N* = 15	*N* = 20	*N* = 25	*N* = 30	*N* = 5	*N* = 10	*N* = 15	*N* = 20	*N* = 25	*N* = 30
Circle shaped	EVR	0.726	0.711	0.708	0.907	0.909	0.926	0.354	0.293	0.473	0.124	0.220	0.083
	EI	**0.826**	0.748	0.860	0.871	0.929	0.911	**0.127**	0.193	0.270	0.032	0.095	**0.032**
	UCB	0.626	**0.804**	0.876	0.925	0.925	0.949	0.396	**0.096**	0.246	0.091	0.155	0.096
	LSE	0.786	0.702	0.840	0.920	0.949	0.961	0.471	0.247	0.095	0.253	0.222	0.071
	ILS-UCB	0.566	0.796	**0.957**	**0.954**	**0.958**	**0.963**	0.555	0.333	**0.065**	**0.085**	**0.016**	0.085
	RASEC	0.553	0.796	0.907	0.926	0.923	0.927	0.445	0.266	0.266	0.276	0.138	0.069
Square shaped	EVR	0.593	0.910	0.912	0.928	0.951	0.960	0.343	0.260	**0.163**	0.234	**0.064**	**0.056**
	EI	**0.808**	0.875	**0.936**	0.924	0.906	0.904	0.507	0.290	0.414	0.352	0.240	0.157
	UCB	0.760	0.845	0.878	0.958	0.945	0.956	0.314	0.330	0.562	0.259	0.262	0.193
	LSE	0.719	0.869	0.896	0.954	0.968	**0.976**	**0.289**	0.356	0.238	**0.193**	0.205	0.178
	ILS-UCB	0.804	0.853	0.922	**0.959**	0.963	0.968	0.399	0.314	0.304	0.338	0.150	0.128
	RASEC	0.794	**0.933**	0.933	0.953	**0.969**	0.974	0.297	**0.148**	0.178	0.187	0.180	0.094
Horseshoe shaped	EVR	0.296	0.329	0.573	0.604	0.613	0.812	0.458	0.751	0.660	0.334	0.328	0.297
	EI	0.393	0.414	0.459	0.470	0.620	0.774	0.235	**0.278**	0.144	**0.161**	0.260	0.129
	UCB	0.480	**0.636**	**0.792**	**0.762**	0.812	0.817	0.376	0.577	0.419	0.221	**0.198**	0.168
	LSE	**0.605**	0.492	0.614	0.737	0.820	0.883	0.442	0.438	0.376	0.313	0.267	0.219
	ILS-UCB	0.527	0.534	0.751	0.755	0.806	0.871	1.113	0.963	0.878	0.697	0.416	**0.111**
	RASEC	0.452	0.497	0.579	0.667	**0.843**	**0.892**	**0.218**	0.509	**0.263**	0.264	0.235	0.175

### Segmentation results

**Fig. 9.**
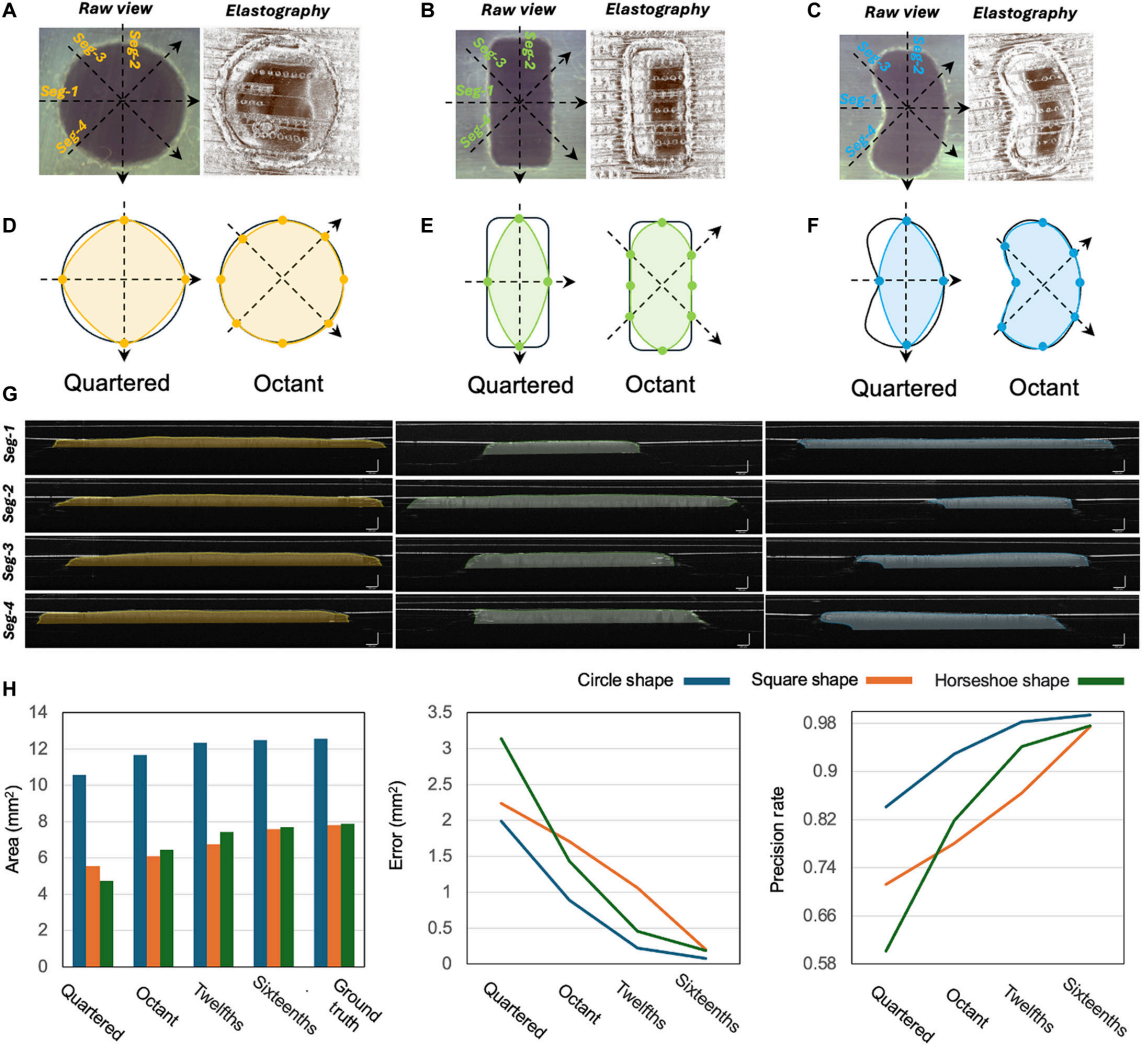
Segmentation methods and results for lesion contouring using sliding B mode. (A) Circular sample (B) rectangular sample, and (C) horseshoe-shaped sample in the original view and elastography, along with their segmentation using the quadrant and octant methods, which display their appearances in both the original view and the elastography images. (D), (E), and (F) illustrate the segmentation of these samples using the quadrant and octant methods, respectively. The bottom part of (G) shows cross-sectional images of the segmented sections. The scale bar in the figure represents 250 μm. (H) compares the area estimation, error, and precision rates with 3 different shapes (circle, square, and horseshoe) using various segmentation methods.

After determining the geometric center of the phantom model, we conducted sliding segmentation experiments based on the sliding B-scan model. This approach enables us to capture variations within the data and enhance our understanding of its geometric properties. Figure 9 presents a comparative analysis of different shape samples: (a) circular, (b) rectangular, and (c) irregular, shown both in their original view and elastography images. Segmentation lines are marked for different segments (Seg-1, Seg-2, Seg-3, and Seg-4). Figure9D to F illustrate the segmentation of these samples using the quartered and octant methods, corresponding to Fig. 9A to C. Specifically, Fig. 9D shows the circular sample segmented into quarters and octants, and Fig.9E and F show the rectangular sample and horseshoe-shaped sample segmented similarly, respectively. Figure 9G provides sliding OCT imaging of the segmented sections labeled Seg-1 through Seg-4 for each sample. These visuals effectively demonstrate the differences in the segmentation and appearance of the variously shaped samples, highlighting the efficacy of the quartered and octant segmentation methods.

Figure 9H presents the quantification results of area estimation, estimation error, and precision rates across 3 distinct shapes, circle, square, and horseshoe, utilizing various segmentation methods. The bar chart compares the estimated area to the ground truth area (12.57 mm^2^ for the circular sample, 7.79 mm^2^ for the rectangular sample, and 7.88 mm^2^ for the horseshoe-shaped sample) regarding the 3 shapes across 4 segmentation methods, quartered, octant, twelfths, and sixteenths, alongside the ground truth values.

After applying the 4 progressive segmentation methods, we observed significant improvements in the segmentation results. For the circular sample, the area error decreased from 1.99 to 0.08 mm^2^, with the accuracy increasing from 0.842 to 0.994. In the case of the square sample, the area error was reduced from 2.25 to 0.2 mm^2^, and accuracy improved from 0.712 to 0.974. The area error decreased from 3.14 to 0.19 mm^2^ for the horseshoe-shaped sample, improving the accuracy from 0.602 to 0.976. These results indicate that the progressive density approach significantly enhances segmentation precision across different sample shapes. This indicates that the proposed segmentation strategy can effectively estimate shapes across a diverse range of simulacrum model forms once the center is identified.

## Conclusion

This work introduces a novel robotic bimodal tactile tomography that integrates Bayesian optimization with OCT-based sensing to profile intracavitary targets. The proposed ElastoSight sensor offers dual-modal functionality, using circumferential B scans for precise localization of tumor centroids and sliding B scans for accurate boundary segmentation. We implemented a Bayesian sequential palpation strategy that achieves remarkable submillimeter localization accuracy, with an error margin of just 0.032 mm within 30 iterations. A programmable segmentation method was applied, yielding a precision rate of 98.3% and a low estimated area error of 0.08 mm^2^ for segmenting tumor boundaries. Experimental validation using various phantom models confirmed the system’s ability to reconstruct lesion geometries while concurrently estimating contact forces by analyzing OCT-derived deformations. Overall, this technology is promising to enhance surgical perception during minimally invasive procedures, particularly in early-stage tumor detection and margin assessment during oncological interventions. This hybrid approach effectively addresses critical limitations inherent in conventional RMIS by enabling the real-time stiffness mapping and visualization of subsurface microstructures. By improving the understanding of tissue mechanics and interaction states, this system could substantially elevate the standards of precision and efficacy in surgical practice. Our current research primarily focuses on developing decision-making algorithms, with ongoing efforts to enhance engineering details. We plan to strengthen these components and overall system performance by integrating a surgical robotic system for real-time point cloud registration and alignment. Our future work includes experiments on autonomous searching in phantom models and dynamic in vivo environments with live animals. Further research will expand their framework to include multimodal signal alignment, depth-resolved elastography for layered tissue characterization, and integration with robotic control mechanisms for closed-loop palpation.

## Data Availability

All data needed to evaluate the conclusions are presented in the article and the Supplementary Materials. Additional data supporting this study’s findings are available from the corresponding authors upon reasonable request.
