# Supplementary material for: Bimodal Tactile Tomography with Bayesian Sequential Palpation for Intracavitary Microstructure Profiling and Segmentation
Source: Cyborg Bionic Syst. 2025 Sep 2;6:0348. doi: 10.34133/cbsystems.0348 (PMC12604559; doi:10.34133/cbsystems.0348)
Supplement: Supplementary 1 — Movies S1 to S3 [file cbsystems.0348.f1.zip › Supplementary Materials.docx]

## Supplementary Materials

**Movie S1.** Principal Demonstration of OCT-based tactile sensors.

**Movie S2**. Visualization results of fruit palpation experiments from the OCT-based tactile sensor.

**Movie S3.** Real-Time Updated Expectation, Sampling, and Location Maps for Center Location Identification in Three Distinct Shapes Using Bayesian Optimization.
